# Poly(ionic liquid)/OPBI Composite Membrane with Excellent Chemical Stability for High-Temperature Proton Exchange Membrane

**DOI:** 10.3390/polym15153197

**Published:** 2023-07-27

**Authors:** Yiming Xiao, Haoran Chen, Ranxin Sun, Lei Zhang, Jun Xiang, Penggao Cheng, Huaiyuan Han, Songbo Wang, Na Tang

**Affiliations:** 1 Department of Chemical Engineering and Material Science, Tianjin University of Science & Technology, Tianjin 300457, China; tjxiaoyiming@tust.edu.cn (Y.X.); chenhr1010@163.com (H.C.); 16602640897@163.com (R.S.); leizhang@tust.edu.cn (L.Z.); jxiang@tust.edu.cn (J.X.); cpg@tust.edu.cn (P.C.); 2 Tianjin Key Laboratory of Brine Chemical Engineering and Resource Eco-Utilization, Tianjin University of Science & Technology, Tianjin 300457, China; 3 College of Chemistry and Chemical Engineering, Zhengzhou Normal University, Zhengzhou 450044, China; 4 State Key Laboratory of Biobased Fiber Manufacturing Technology, Tianjin University of Science & Technology, Tianjin 300457, China

**Keywords:** poly(ionic liquid), polybenzimidazole, chemical stability, HT-PEM

## Abstract

Despite the outstanding proton conductivity of phosphoric acid (PA)-doped polybenzimidazole (PBI) membranes as high-temperature proton exchange membranes (HT-PEMs), chemical stability is a critical issue for the operation life of PEM fuel cells (PEMFCs). Herein, we introduced polymerized [HVIM]H_2_PO_4_ ionic liquids (PIL) into an OPBI membrane to accelerate proton transfer and enhance the chemical stability of the membrane. Based on the regulation of the intrinsic viscosity of PIL, the entanglement between PIL chains and OPBI chains is enhanced to prevent the loss of PIL and the oxidative degradation of membrane materials. The PIL/OPBI membrane with the intrinsic viscosity of 2.34 dL·g^−1^ (2.34-PIL/OPBI) exhibited the highest proton conductivity of 113.9 mS·cm^−1^ at 180 °C, which is 3.5 times that of the original OPBI membrane. The 2.34-PIL/OPBI membrane exhibited the highest remaining weight of 92.1% under harsh conditions (3 wt% H_2_O_2_; 4 ppm Fe^2+^ at 80 °C) for 96 h, and a much lower attenuation amplitude than the OPBI did in mechanical strength and proton conductivity performance. Our present work demonstrates a simple and effective method for blending PIL with OPBI to enhance the chemical durability of the PA-PBI membranes as HT-PEMs.

## 1. Introduction

Hydrogen energy has drawn attention due to its unique advantages over traditional fossil fuels, including zero emissions and high energy density [1,2,3]. As one of the terminals of hydrogen energy, PEMFC has the characteristics of safety, efficiency, and cleanliness [4,5,6]. Compared to the traditional low-temperature PEMFC (LT-PEMFC) operating at temperatures below 100 °C, the high-temperature PEMFC (HT-PEMFC) operating at 100–200 °C has been widely studied for its advantages of high CO resistance, high electrode kinetics and facilitated water/heat management [7]. As a major element of the HT-PEMFC, HT-PEM should meet the demands of high anhydrous proton conductivity, reasonable mechanical strength, and excellent thermal and oxidative stability [8]. The PA-PBI membrane is has the most potential as a HT-PEM due to its ability to achieve rapid proton transfer between PA and a polymer at high temperatures and without water [9,10]. The acid doping level (ADL) represents the number of PA molecules corresponding to one repeating unit in the polymer. Generally, a higher ADL can accelerate proton conduction in PA-PBI membranes, but it will significantly reduce the mechanical properties of the membrane [11].

Strong redox reactions occur during the operation of PEMFC, so proton exchange membranes need high chemical stability to prevent the oxidative degradation of polymers. Free radicals (e.g., •OH and •OOH) generated in the electrochemical process are the major cause of chemical decomposition of PEM and result in reduced PEMFC performance [12,13,14,15]. To improve the oxidation stability of PEMs under PEMFC operating conditions, a large amount of research has been devoted to improving the antioxidant degradation of PEM to ensure the long-term operation of PEMFC. Hao et al. [16]. reported that doping cerium dioxide nanoparticles can reduce the chemical degradation of PBI membranes and improve the life of fuel cells. Wang et al. [17]. reported that grafting free radical scavengers on the backbone of polyarylethersulfone (PAES) significantly improved the chemical durability of PAES membranes and delayed the degradation of membranes. In addition, some inorganic antioxidants (such as transition metal ions, nanoparticles, and metal oxides) [18,19,20,21,22,23,24,25,26] and organic antioxidants (such as phenols, anthraquinones, and flavonoids) [27,28,29,30] are used in the preparation of composite membranes to slow down their chemical degradation. However, the performance of these composite membranes may decline during fuel cell operation due to the oxidative degradation of antioxidants themselves and poor compatibility with polymers.

In recent years, ionic liquid (IL) has been widely used in fuel cells because of their unique properties such as their high thermostability, wide electrochemical window and excellent ionic conductivity [31,32,33,34,35]. The IL/PBI membrane obtained via simple blending exhibits superior proton conductivity, but the leaching and oxidation of IL can cause a decrease in membrane stability. At present, the introduction of ionic liquids as fillers into PBI membranes is carried out mainly to improve the proton conductivity of a membrane [36,37,38], but there are almost no relevant literature reports on using ionic liquids to enhance membrane chemical stability. For PEMFCs, stability is one of the main technical challenges dominating the field and is crucial for practical applications [39,40,41,42,43]. The macromolecular polymeric ionic liquid (PIL) polymerized by IL can form a linear arrangement of proton acceptors and proton donors to accelerate proton transport while preventing loss and oxidation in the membrane [38,44,45,46].

In this work, we introduce synthesized PIL into [2,2-(p-oxydiphenylene)-5,5-bibenzimidazole] (OPBI) to prepare PIL/OPBI hybrid membranes. PIL accelerate proton transfer by acting as additional proton conductors, and prevent the oxidative degradation of the membrane by polymerizing into macromolecular chains to increase chemical stability. [HVIM]H_2_PO_4_ ionic liquids with the cation and anion of vinyl imidazole and dihydrogen phosphate, respectively, were synthesized via alkylation and an anion exchange reaction. PIL (P[HVIM]H_2_PO_4_) were synthesized via the polymerization of double bond groups in [HVIM]H_2_PO_4_. The PIL/OPBI membranes with varying degrees of polymerization of P[HVIM] H_2_PO_4_ were prepared to investigate PA doping levels (ADL), mechanical properties, and proton conductivity. To further explore the chemical durability of the hybrid membrane, the weight retention and proton conductivity of the membrane after long-term immersion in Fenton’s reagent were measured.

## 2. Materials and Methods

### 2.1. Materials

Briefly, 3,3′-diaminobenzidine (99 wt%), 4,4′-diphenyl ether dicarboxylic acid (98 wt%), N-vinylimidazole (99 wt%), and phosphorus pentoxide (98.5 wt%) were obtained from Shanghai Aladdin Biochemical Technology Co., Ltd. (Shanghai, China). Ethane bromide (99 wt%), ferrous sulfate (99 wt%), ethyl acetate (99.7 wt%), and phosphoric acid (85 wt%) were obtained from Tianjin Damao Chemical Reagent Factory (Tianjin, China). Azobisisobutyronitrile (98 wt%), anhydrous ethanol (99.5 wt%) N,N-Dimethylformamide (99 wt%), and sodium chloride (99.5 wt%) were obtained from Tianjin Jiangtian Chemical Technology Co., Ltd. (Tianjin, China). Hydrogen peroxide (3 wt%) was purchased from Shandong Likang Medical Technology Co., Ltd. (Taian, China).

### 2.2. Synthesis of the [HVIM]H_2_PO_4_

**Scheme 1:**
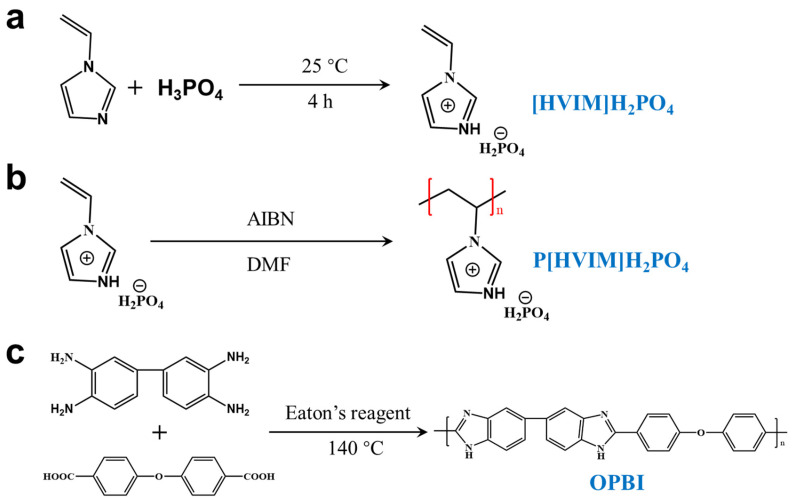
Preparation of [HVIM]H_2_PO_4_ (**a**), P[HVIM]H_2_PO_4_ (**b**), and OPBI (**c**).

As shown in Scheme 1a, 28.24 g of 1-vinylimidazole (0.3 mol) was added to a three-necked flask, and then 36 mL of 0.01 mol/L phosphoric acid solution was slowly added dropwise under ice-water-bath conditions. After stirring at room temperature for 4 h, the reactive yellow liquid was distilled under reduced pressure at 80 °C to remove solvent water. Further, the cream-white viscous liquid product [HVIM]H_2_PO_4_ (94% yield) was obtained after washing with ethyl acetate for several times and drying at 40 °C in a vacuum for 24 h.

### 2.3. Synthesis of P[HVIM]H_2_PO_4_

P[HVIM]H_2_PO_4_ were synthesized via the free-radical polymerization of double bonds in [HVIM]H_2_PO_4_, as shown in Scheme 1b. Briefly, 19.2 g of synthesized [HVIM]H_2_PO_4_ (0.1 mol) was dissolved in 500 mL of DMF solvent to prepare a certain concentration reaction system in a three-necked flask. Then, the initiator azodiisobutyronitrile (AIBN) (0.192 g, 1% of the total mass of monomers) was added and heated under N_2_ protection for a reflux reaction for 24 h. The reaction mixture was centrifuged. A pale-white product P[HVIM]H_2_PO_4_ (95% yield) was obtained by washing the solid phase several times with DMF and absolute ethanol, followed by vacuum drying for 24 h at 60 °C.

### 2.4. Preparation of P[HVIM]H_2_PO_4_/OPBI Membrane

As shown in Scheme 1c, the OPBI polymer with an intrinsic viscosity of 1.55 dL g^−1^ (0.5 g dL^−1^ DMSO, 30 °C) was synthesized in accordance with our previous report [47,48,49]. OPBI/DMSO and P[HVIM]H_2_PO_4_/DMSO solutions were obtained by adding 0.6 g of the OPBI polymer and 0.09 g of P[HVIM]H_2_PO_4_ into 30 mL of DMSO and stirring for 12 h. The P[HVIM]H_2_PO_4_/OPBI/DMSO casting solution was prepared by mixing and stirring the above two solutions for 5 h and ultrasonic stirring for 0.5 h. The casting solution was poured onto a clean glass plate for tape casting, followed by drying at 80 °C for 12 h to evaporate the solvent and solidify it into a membrane. The membrane was removed from the glass plate and dried in a vacuum oven at 100 °C for 24 h to prepare the x-PIL/OPBI membrane, where x represents the intrinsic viscosity of P[HVIM]H_2_PO_4_. IL/OPBI membranes were prepared by blending [HVIM]H_2_PO_4_ with OPBI in accordance with the above method.

### 2.5. Measurements

The Fourier transform infrared (FTIR, Bruker TENSOR27, Billerica, MA, USA) spectra of the polymers and membranes were determined in the peak range of 4000 to 700 cm^−1^. The ^1^H NMR (Bruker AV III 400) spectra wEre measured using deuterated dimethyl sulfoxide (D6-DMSO) as the solvent and tetramethylsilane (TMS) as an internal standard. The thermal gravimetric analysis (TGA, TA Q50, TA Instruments, New Castle, DE, USA) of membranes was performed in the temperature range of 80 to 750 °C under a nitrogen atmosphere. The mechanical properties of the membranes were tested using 11-3 5KN apparatus (L&W, Stockholm, Swedish).

### 2.6. Intrinsic Viscosity

The intrinsic viscosity of the polymers was measured using an Ubbelohde viscometer at 30 °C in DMSO (0.5 g dL^−1^) to characterize the relative molecular weight. Firstly, the efflux time of the DMSO solvent (*t*_0_, s) and polymer solution (*t*, s) was measured using the Ubbelohde viscometer. Then, the relative viscosity (*η_r_*) and increasing specific viscosity (*η_sp_*) were calculated according to the equations *η_r_* = *t*/*t*_0_ and *η_sp_* = *η_r_* − 1, respectively. The intrinsic viscosity ([*η*]) of OPBI and PIL was calculated using the following formula:

(1) $$\left[\eta \right]=\frac{1}{c}\sqrt{2(\eta_{sp}-\ln\eta_{r})}$$

where c is the concentration of polymer solution, g/dL.

### 2.7. PA Doping Performance

The membrane sample was cut into 50 mm × 20 mm rectangular pieces and dried. Then, after soaking it in 85 wt% PA solution at 100 °C for 72 h, the weight and volume of the membrane sample before and after PA doping were recorded.

The *ADL* of the membranes was calculated using the following formula:

(2) $$ADL=\frac{{(W}_{doped}-W_{undoped})/M_{PA}}{\left[W_{undoped}\times \left(1-X\%\right)\right]/M_{OPBI}}$$

where *X*% represent the mass percentage of *IL* or PIL in the membrane sample. *M_PA_* and *M_OPBI_* represent the molecular weights of *PA* and *OPBI* repeat units, respectively. *W_undoped_* and *W_doped_* represent the mass of membrane before and after *PA* immersion, respectively.

The volume swelling ratio of the membranes was calculated using the following formula:

(3) $$Swelling\;ratio=\frac{\left(V_{wet}-V_{dry}\right)}{V_{dry}}\times 100\%$$

where *V_dry_* and *V_wet_* represent the volume of the membranes before and after immersion in *PA*, respectively.

### 2.8. Oxidation Stability

The membrane samples were cropped to 30 mm × 15 mm and soaked in deionized water at 80 °C for 24 h to remove the residual solvent. The membrane samples were dried and immersed in Fenton’s reagent (4 ppm Fe^2+^ in 3 wt% H_2_O_2_) at 80 °C. The membrane samples were taken out and dried regularly, and the remaining weight was recorded to evaluate oxidation stability. In order to avoid the deviation of the test results of membrane oxidation stability caused by the loss of the ionic liquid in Fenton’s reagent, the mass loss of membrane due to oxidation is calculated by subtracting the mass loss of the ionic liquid from the mass loss of the membrane in Fenton’s reagent. The remaining weight of membranes after Fenton’s reagent treatment was calculated using the following formula:

(4) $$Remaining\;weight\left(\%\right)=\frac{W_{i}+W_{IL}}{W_{0}}\times 100\%$$

where *W*_0_ and *W_i_* represent the mass of the membranes before and after immersion in Fenton’s reagent, respectively, and *W_IL_* represents the mass of *IL* loss. *W_IL_* was obtained from the blank test, which measured the loss mass of the membrane in distilled water at 80 °C at the same time.

### 2.9. Doping Stability of PA and IL

The PA-doped membrane samples were cured at 90% RH and 80 °C, and the stability of *PA* doping was evaluated by recording the quality changes of the membrane samples after curing for different times. The loss rate of PA was calculated using the following formula:

(5) $$The\;loss\;rate\;of\;PA\left(\%\right)=\frac{W_{0}-W_{i}}{W_{PA}}\times 100\%$$

where *W_0_* and *W_i_* represent the mass of the membranes before and after treatment, respectively, and *W_PA_* represents the mass of *PA* in the membranes.

The membrane samples were immersed in distilled water at room temperature, and the stability of ionic liquid doping was evaluated by recording the mass changes of the membrane samples after immersion for different times. The loss rate of IL was calculated using the following formula:

(6) $$The\;loss\;rate\;of\;IL\left(\%\right)=\frac{W_{0}-W_{i}}{W_{IL}}\times 100\%$$

where *W_0_* and *W_i_* represent the mass of the membranes before and after immersion, respectively, and *W_IL_* represents the mass of *IL* in the membranes.

### 2.10. Proton Conductivity and Fuel Cell Performance

The proton conductivity (σ, S cm^−1^) at 80~180 °C without additional humidification was determined via the membrane resistance (R and Ω). Electrochemical impedance spectroscopy was performed with two electrodes on a Bio Logic SP-300 electrochemical workstation, and the test frequency range was 1~10^5^ Hz. The proton conductivity of PA-doped membranes was calculated using the following formula:

(7) $$\sigma (S\;{cm}^{-1})=\frac{L}{R\times A}$$

where *L* is the distance between the two electrodes, *R* is the measured resistance, and *A* is the cross-sectional area of the membranes.

Two pieces of commercial carbon paper loaded with a Pt catalyst (0.6 mg cm^−2^) were sandwiched on both sides of the membrane sample to produce a membrane electrode assembly (MEA) with an active electrode area of 2.0 × 2.0 cm^2^. H_2_ and O_2_ were introduced from the anode and cathode at a flow rate of 200 mL min^−1^, respectively, and the polarization curves of MEA were measured using the current step potential method at 160 °C.

## 3. Results and Discussion

### 3.1. Synthesis of the PIL/OPBI Membrane

**Figure 1:**
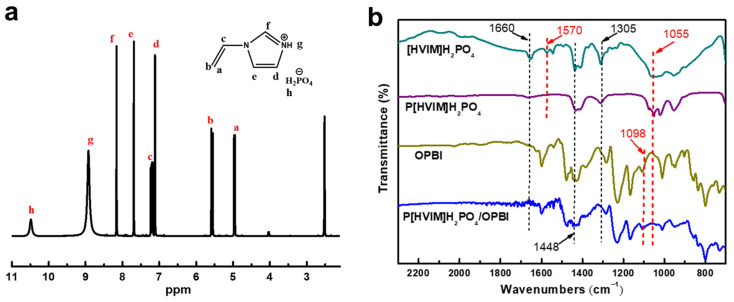
The ^1^H NMR spectra of [HVIM]H_2_PO_4_ (**a**); the FTIR spectra of [HVIM]H_2_PO_4_, P[HVIM]H_2_PO_4,_ and all the membranes (**b**).

Figure 1a shows the ^1^H NMR spectrum of the synthesized [HVIM]H_2_PO_4_. The hydrogen chemical shifts (d, e, and f) on the imidazole ring in the [HVIM]H_2_PO_4_ cation are assigned to peaks at 7.1, 7.7, and 8.2 ppm, respectively. The hydrogen chemical shifts in the vinyl group (a, b, and c) of the imidazole ring side chain are assigned to the peaks at 5.0, 5.6, and 7.2 ppm, respectively. The hydrogen chemical shift (g) on the imine group in the imidazole ring after ionization is assigned to the peak at 9.0 ppm. The hydrogen chemical shift on H_2_PO_4_^−^ (h) is assigned to peaks at 10.5 ppm. The hydrogen in [HVIM]H_2_PO_4_ corresponds well to the characteristic peaks of hydrogen in the ^1^H NMR spectrum, which verifies the chemical structure of the synthesized [HVIM]H_2_PO_4_. The FT-IR spectra of [HVIM]H_2_PO_4_, P[HVIM]H_2_PO_4_, OPBI and P[HVIM]H_2_PO_4_/OPBI are shown in Figure 1b. All samples exhibit characteristic peaks at 1660 cm^−1^, 1448 cm^−1^ and 1305 cm^−1^, corresponding to the C=N bond, C-N bond, and imidazole ring, which proved the imidazole ring structure in IL, PIL and OPBI [49]. Compared to [HVIM]H_2_PO_4_, the C=C bond characteristic peak at 1570 cm^−1^ does not appear in P[HVIM]H_2_PO_4_, indicating the generation of C=C bond polymerization. The intrinsic viscosity of P[HVIM]H_2_PO_4_ was further measured at 30 °C in 0.5 g/L DMSO to demonstrate the degree of polymerization of P[HVIM]H_2_PO_4_. As shown in Figure S1, the intrinsic viscosity of P[HVIM]H_2_PO_4_ was regulated by the polymerization temperature and concentration. Briefly, 0.59-PIL/OPBI, 1.27-PIL/OPBI, and 2.34-PIL/OPBI membranes were obtained by blending OPBI and P[HVIM]H_2_PO_4_ with three intrinsic viscosities (0.59 dL·g^−1^, 1.27 dL·g^−1^, and 2.34 dL·g^−1^), respectively. P[HVIM]H_2_PO_4_/OPBI membrane shows the characteristic peak of the Ar-O-Ar bond in OPBI at 1098 cm^−1^, and the characteristic peak of P=O bond in P[HVIM]H_2_PO_4_ at 1055 cm^−1^, which proves that P[HVIM]H_2_PO_4_ successfully blended into OPBI.

### 3.2. Thermal Stability

**Figure 2:**
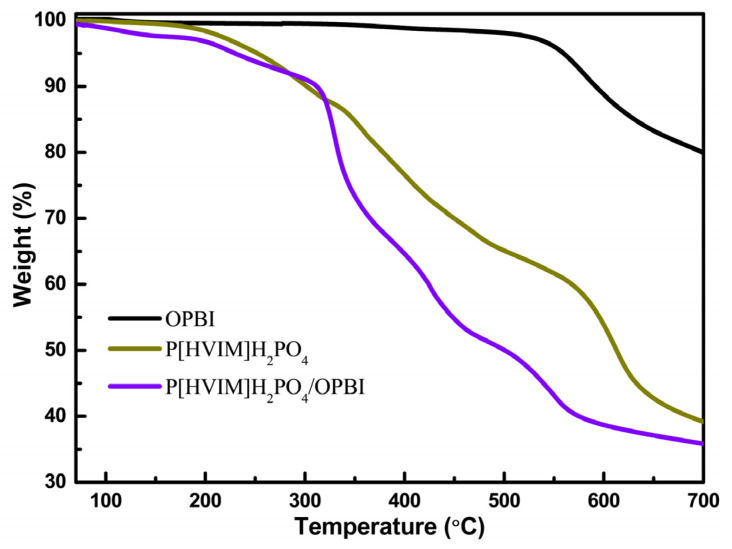
TGA curves of OPBI, P[HVIM]H_2_PO_4_, and P[HVIM]H_2_PO_4_/OPBI.

Figure 2 presents the TGA curves of OPBI, P[HVIM]H_2_PO_4_, and P[HVIM]H_2_PO_4_/OPBI. The mass loss of P[HVIM]H_2_PO_4_/OPBI and OPBI membranes occurs at approximately 500 °C, due to the thermal degradation of the OPBI polymer backbone. In addition, P[HVIM]H_2_PO_4_ and the P[HVIM]H_2_PO_4_/OPBI membrane exhibit weight loss at about 200 °C and 300 °C, corresponding to the dehydration polymerization of anionic H_2_PO_4_^−^ and the decomposition of the cationic imidazole ring, respectively [50,51,52]. The mass loss of the P[HVIM]H_2_PO_4_/OPBI membrane below 200 °C is less than 5%, indicating that the membrane has good thermal stability to withstand the operating temperature of 100–200 °C as HT-PEM.

### 3.3. PA Doping Behaviors and Mechanical Strength

**Figure 3:**
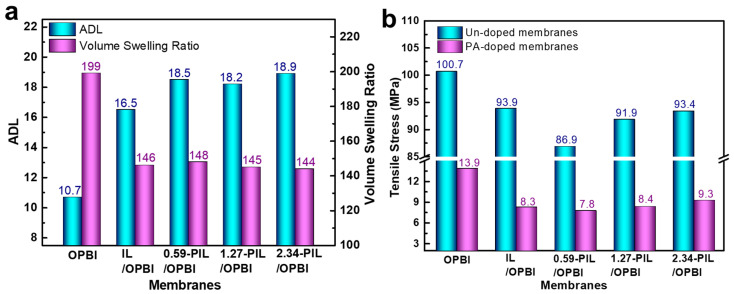
ADL (**a**), volume swelling ratio (**a**), and tensile strength (**b**) of PIL/OPBI membranes.

Figure 3a shows the ADL of the membranes after soaking them in 85 wt% PA at 100 °C for 72 h. The ADLs of OPBI and IL/OPBI membranes are 10.7 and 16.5, respectively. The ADL of IL/OPBI membrane is 1.5 times that of the original OPBI membrane, which is attributed to the imidazole ring cation with the Bronsted base effect in IL. The ADLs of the PIL/OPBI membranes with different intrinsic viscosities of PIL are about 18.5, which was increased by 12% compared to that of IL/OPBI membranes, indicating that the PIL arranged in a chain structure were conducive to the adsorption of more PA. Although the ADLs of IL/OPBI and PIL/OPBI membranes are significantly higher than those of the OPBI membranes, the volume swelling ratio of PA-doped IL/OPBI and PIL/OPBI membranes is only about 73% of that of OPBI membranes, which is due to the imidazole ring in ionic liquids providing more space sites for PA doping to improve the volumetric stability of the blend membranes [8,46,53].

As shown in Figure 3b, the tensile strength of PA-undoped OPBI, IL/OPBI, 0.59-PIL/OPBI, 1.27-PIL/OPBI, and 2.34PIL/OPBI membranes are 100.7 MPa, 93.9 MPa, 86.9 MPa, 91.9 MPa, and 93.4 MPa, respectively. Compared to that of the OPBI membrane, the tensile strength of each blend membrane is slightly lower, as the ionic liquids in the membrane reduce the tight arrangement structure of the polymer chains. Due to the plasticization effect of PA [39,42,43], the tensile strength of all membranes decreased significantly after immersion in PA. The mechanical strength of PA-doped IL/OPBI and PIL/OPBI membranes with higher ADLs decreases more significantly. In addition, the mechanical strength of PIL/OPBI membranes increases slightly with the increase in the intrinsic viscosity of PIL. The tensile strength of the 2.34 PIL/OPBI membrane is increased by 19% compared to the 0.59-PIL/OPBI membrane, indicating that the increase in the polymerization degree of PIL can enhance the mechanical properties of the blend membrane by increasing the mechanical strength of PIL.

### 3.4. Proton Conductivity

**Figure 4:**
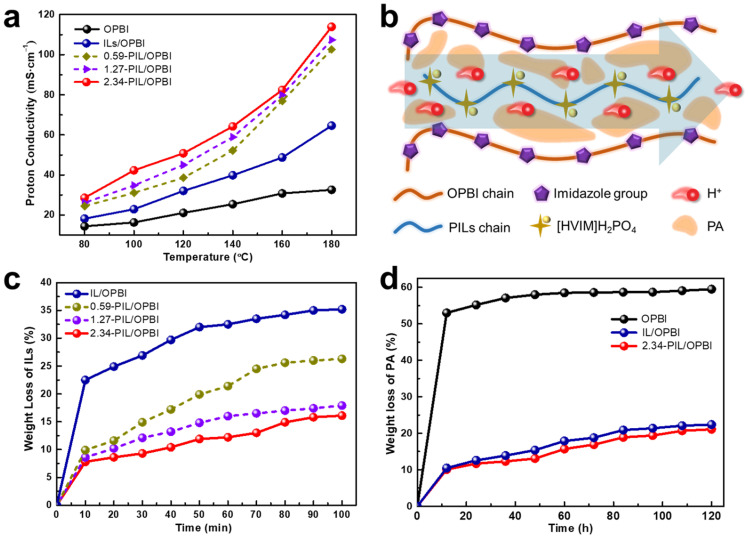
Proton conductivity (**a**) of PIL/OPBI membranes; proton transport schematic (**b**) of PIL/OPBI membrane; weight loss of IL (**c**) and PA (**d**) of IL/OPBI and PIL/OPBI membranes.

Figure 4a exhibits the proton conductivity of PA-doped blend membranes at 80–180 °C without humidification. The proton conductivity of all membranes increases with temperature, which is attributed to the acceleration of proton transfer kinetics. The proton conductivity of OPBI, IL/OPBI, 0.59-PIL/OPBI, 1.27-PIL/OPBI, and 2.34-PIL/OPBI membranes at 180 °C are 32.6 mS·cm^−1^, 64.6 mS·cm^−1^, 102.6 mS·cm^−1^, 107.4 mS·cm^−1^ and 113.9 mS·cm^−1^, respectively. The proton conductivity of IL/OPBI membranes at 180 °C is 2.0 times that of OPBI membranes, which is attributed to the higher ADL and additional IL proton conductors in IL/OPBI membranes. Compared to that of IL/OPBI, the proton conductivity of the 0.59-PIL/OPBI membrane increased by 59% at 180 °C, indicating that the polymerization of IL can significantly accelerate proton transfer in the membrane. In addition, the increase in the polymerization degree of PIL can further accelerate proton transfer in PIL/OPBI membranes. The proton conductivity of the 2.34-PIL/OPBI membrane at 180 °C is 3.5 times, 1.8 times and 1.1 times that of OPBI, IL/OPBI and 0.59-PIL/OPBI membranes, respectively. As shown in Figure 4b, ionic liquid groups on PIL with a long-molecular-chain arrangement and the adsorbed PA synergistically construct continuous proton channels to accelerate proton transfer in the PIL/OPBI membrane. As shown in Figure 4c, the IL loss rate of IL/OPBI and PIL/OPBI membranes in water at 30 °C was investigated. After 100 min, the IL loss rates of the IL/OPBI, 0.57-PIL/OPBI, 1.27-PIL/OPBI, and 2.34-PIL/OPBI membranes were 35.2%, 26.3%, 19.7%, and 16.1%, respectively. The IL loss rate of the 2.34-PIL/OPBI membrane is only 46% and 61% of that of IL/OPBI and 0.57-PIL/OPBI membranes, indicating that the polymerization of IL and the increase in the PIL polymerization degree could significantly reduce the loss of IL in the membrane. Compared to small-molecule IL, long-chain PIL are more easily entangled with OPBI polymers, thereby increasing the stability of PIL in the membrane. As shown in Figure 4d, the PA loss rates of the OPBI, IL/OPBI and 2.34-PIL/OPBI membranes after 120h at 90 °C and 90% RH are 59.5%, 22.4% and 21.1%, respectively, suggesting that the introduction of IL and PIL can increase the stability of PA.

### 3.5. Fuel Cell Performance

**Figure 5:**
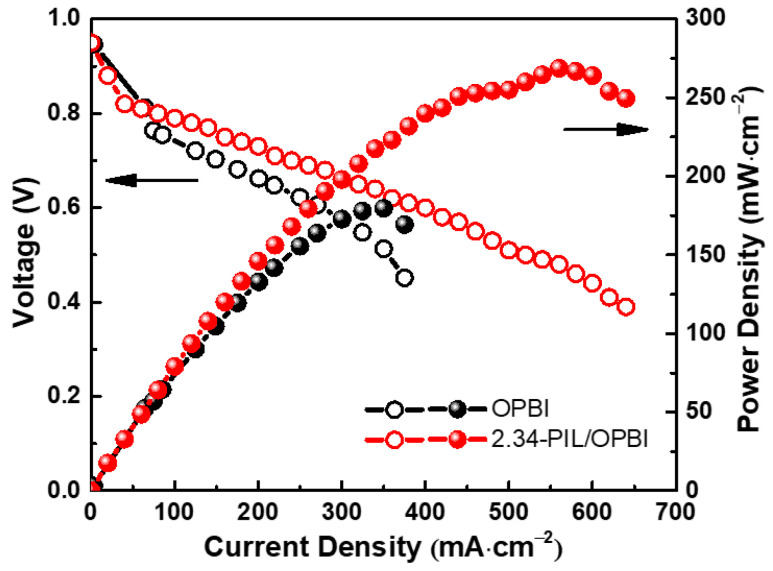
Polarization and power density curves of the MEAs based on OPBI, and 2.34-PIL/OPBI membranes.

Figure 5 shows the polarization curves (open symbols) and power density curves (filled symbols) of MEAs based on the PA-doped OPBI and 2.34-PIL/OPBI membranes. The open circuit voltages of the OPBI and 2.34-PIL/OPBI membranes are 0.94 V and 0.95 V, respectively, which are greater than 0.9 V, indicating that the membranes have good compactness to prevent gas crossover during cell operation. The peak power densities of MEAs based on the OPBI and 2.34-PIL/OPBI membranes are 180 and 269 mW cm^−2^, respectively, and the corresponding current densities are 350 and 560 mA cm^−2^, respectively. Obviously, the fuel cell based on the 2.34-PIL/OPBI membrane exhibits a higher peak power density, which is 1.5 times higher than that based on the OPBI membrane. More PA and proton conductor PIL make the PIL/OPBI membrane have higher proton conductivity to ensure excellent fuel cell performance.

### 3.6. Oxidation Stability

**Figure 6:**
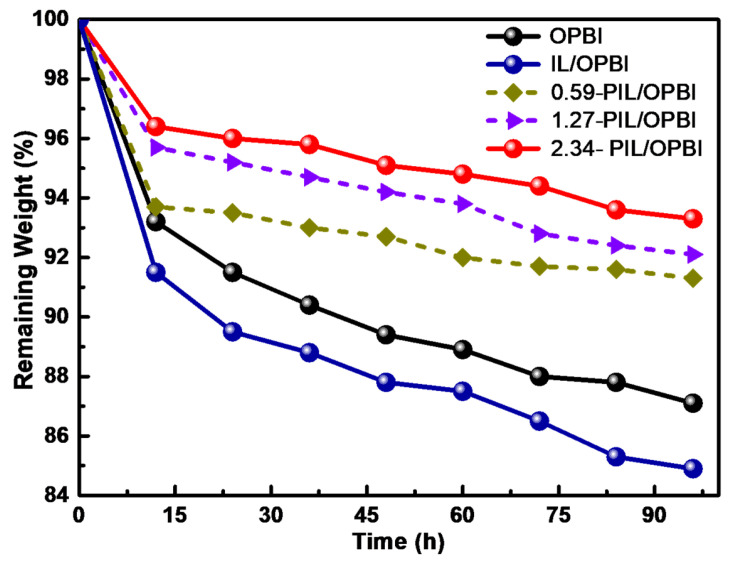
Remaining weight of OPBI, IL/OPBI and PIL/OPBI membranes after Fenton’s reagent (H_2_O_2_ 3 wt%, Fe^2+^ 4 ppm) treatment.

As shown in Figure 6, the weight of all membranes gradually decreased with the increasing soaking time in Fenton’s reagent (H_2_O_2_ 3 wt%, Fe^2+^ 4 ppm) at 80 °C, due to the oxidative degradation by •OH radicals in Fenton’s reagent. The residual weights of the OPBI, IL/OPBI, 0.59-PIL/OPBI, 1.27-PIL/OPBI, and 2.34-PIL/OPBI membrane after 96 h were 87.1%, 84.9%, 91.3%, 92.1%, and 93.3%, respectively. Among them, the residual weight of IL/OPBI membrane was less than that of the OPBI membrane, suggesting that small-molecule IL were more easily oxidized and degraded than was the OPBI polymer. Compared to the OPBI and IL/OPBI membranes, the residual weight of the PIL/OPBI membranes increase significantly, and increase with the increase in the intrinsic viscosity of PIL. The macromolecular-chain PIL increase the oxidative stability of IL and prevent the degradation of OPBI by intertwining with the OPBI polymer chain, thus increasing the chemical stability of PIL/OPBI membranes.

**Figure 7:**
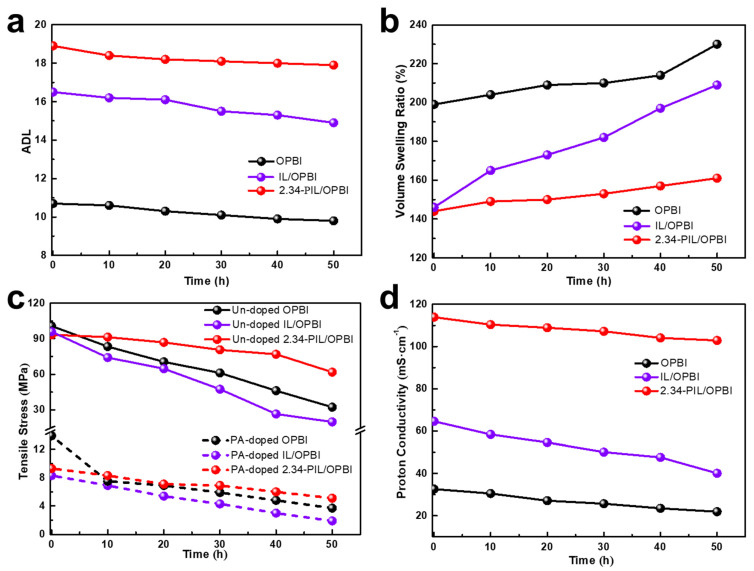
ADL (**a**), volume stability (**b**), mechanical strength (**c**), and proton conductivity at 180 °C (**d**) of OPBI, IL/OPBI and 2.34-PIL/OPBI membranes in Fenton’s reagent for different times.

To further evaluate the effect of oxidative stability on the other properties of the membranes, Figure 7 shows the ADL, volume stability, mechanical strength and proton conductivity of the OPBI, IL/OPBI and 2.34-PIL/OPBI membranes in Fenton’s reagent for different times. The performance of each membrane is attenuated with the increase in the soaking time, which is attributed to the oxidative breakdown of the membrane structure. The ADL of the OPBI, IL/OPBI and 2.34-PIL/OPBI membranes after 50 h decreased by 9%, 10% and 5%, the volume swelling ratio increased by 16%, 43% and 12%, the tensile strength (PA doped) decreased by 73%, 77% and 45%, and the proton conductivity at 180 °C decreased by 32%, 38% and 10%, respectively. Obviously, the 2.34-PIL/OPBI membrane had better performance stability during the oxidation treatment, proving that the introduction of high-intrinsic-viscosity PIL into a OPBI membrane can significantly enhance the chemical stability of the membrane.

## 4. Conclusions

**Figure 8:**
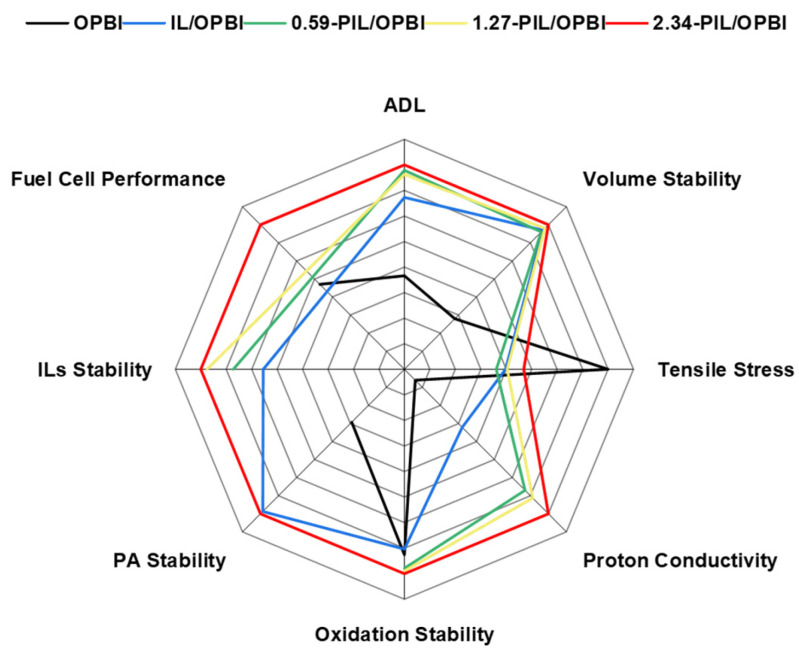
Comparison of performance between OPBI, IL/OPBI, and x-IL/OPBI membranes.

In conclusion, we report a simple and effective method of polymerization of IL to prepare PIL/OPBI blend membranes, which can significantly increase ADL and reduce the volume swelling ratio. The intrinsic viscosity of PIL is regulated by the reaction temperature and concentration. More importantly, the formation of macromolecular-chain PIL accelerates the transfer of protons in the membrane, while preventing the loss of PIL and enhancing oxidative stability through the entanglement between PIL and OPBI chains. As shown in Figure 8, the 2.34-PIL/OPBI membrane is far superior to both the OPBI and IL/OPBI membranes, with excellent proton conductivity and significant PA and IL retention. Meanwhile, the 2.34-PIL/OPBI membrane exhibits excellent fuel cell performance, with a peak power density of 257 mW cm^−2^ at 160 °C. The results of this study indicate the significant potential of the PIL/OPBI composite membrane in enhancing the chemical stability of PA-PBI membranes as PEMs by regulating the molecular chain of PIL.

## Data Availability

All data are included within this manuscript.

## Supplementary Materials

The following supporting information can be downloaded at: https://www.mdpi.com/article/10.3390/polym15153197/s1, Figure S1: The effect of reaction temperature (a) and reaction concentration (b) on the inherent viscosity of P[HVIM]H_2_PO_4_.
