# Supplementary material for: Poly(ionic liquid)/OPBI Composite Membrane with Excellent Chemical Stability for High-Temperature Proton Exchange Membrane
Source: Polymers (Basel). 2023 Jul 27;15(15):3197. doi: 10.3390/polym15153197 (PMC10421078; doi:10.3390/polym15153197)
Supplement: Supplementary file 1 [file polymers-15-03197-s001.zip › polymers-2476106-supplementary.pdf]

# Supporting Information

# Poly(ionic liquid)/OPBI Composite Membrane with Excellent Chemical Stability for High-Temperature Proton Exchange Membrane

## 1. The intrinsic viscosity of P[HVIM]$H_2PO_4$

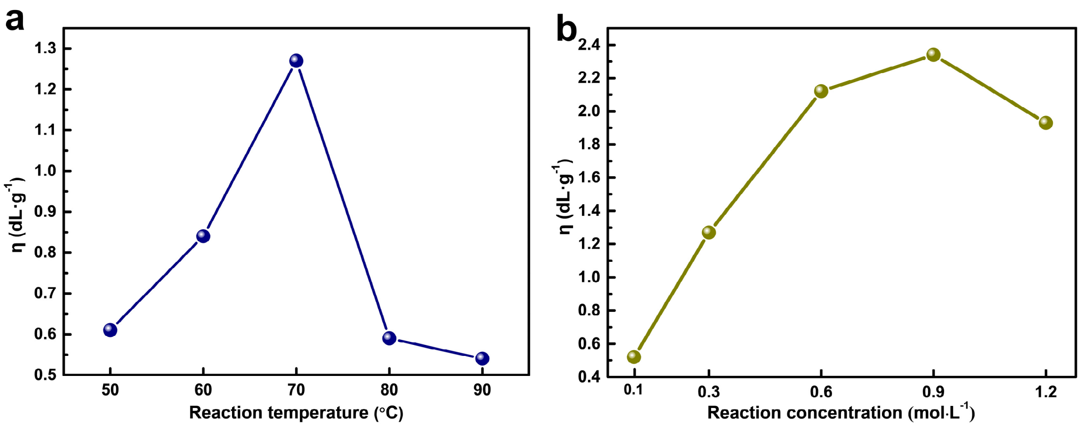


**Figure S1.** The effect of reaction temperature (a) and reaction concentratiuon (b) on the inherent viscosity of P[HVIM]$H_2PO_4$
